# The Echocardiographic Parameters of Systolic Function Are Associated with Specific Metabolomic Fingerprints in Obstructive and Non-Obstructive Hypertrophic Cardiomyopathy

**DOI:** 10.3390/metabo11110787

**Published:** 2021-11-18

**Authors:** Martino Deidda, Antonio Noto, Daniele Pasqualucci, Claudia Fattuoni, Luigi Barberini, Cristina Piras, Pier Paolo Bassareo, Maurizio Porcu, Giuseppe Mercuro, Christian Cadeddu Dessalvi

**Affiliations:** 1 Department of Medical Sciences and Public Health, University of Cagliari, 09124 Monserrato, Italy; barberini@unica.it (L.B.); giuseppemercuro@gmail.com (G.M.); cadedduc@unica.it (C.C.D.); 2 Department of Biomedical Sciences, University of Cagliari, 09124 Monserrato, Italy; antonionoto@unica.it (A.N.); cristina.piras@unica.it (C.P.); 3 Cardiothoracic Unit, Azienda Ospedaliera Brotzu, 09134 Cagliari, Italy; Pasqualucci.Daniele@gmail.com (D.P.); maurizioporcu@aob.it (M.P.); 4 Department of Chemical and Geological Sciences, University of Cagliari, 09124 Monserrato, Italy; cfattuon@unica.it; 5 School of Medicine, Mater Misericordiae University Hospital and Our Lady’s Children’s Hospital Crumlin, University College of Dublin, D07 R2WY Dublin, Ireland; piercard@inwind.it

**Keywords:** metabolomics, global longitudinal strain, 3D echocardiography, obstructive hypertrophic cardiomyopathy, non-obstructive hypertrophic cardiomyopathy

## Abstract

The purpose of this study was to assess whether metabolomics, associated with echocardiography, was able to highlight pathophysiological differences between obstructive (OHCM) or non-obstructive (NOHCM) hypertrophic cardiomyopathy. Thirty-one HCM patients underwent standard and advanced echocardiography; a plasma sample was collected for metabolomic analysis. Results. Patients with OHCM compared with subjects with NOHCM had higher values of 2DLVEF (66.5 ± 3.3% vs. 60.6 ± 1.8%, *p* < 0.01), S wave (7.6 ± 1.1 vs. 6.3 ± 0.7 cm/s, *p* < 0.01) and 3D global longitudinal strain (17.2 ± 4.2%, vs. 13.4 ± 1.3%, *p* < 0.05). A 2-group PLS-Discriminant Analysis was performed to verify whether the two HCM groups differed also based on the metabolic fingerprint. A clear clustering was shown (ANOVA *p* = 0.014). The most discriminating metabolites resulted as follows: in the NOHCM Group, there were higher levels of threitol, aminomalonic acid, and sucrose, while the OHCM Group presented higher levels of amino acids, in particular those branched chains, of intermediates of glycolysis (lactate) and the Krebs cycle (fumarate, succinate, citrate), of fatty acids (arachidonic acid, palmitoleic acid), of ketone bodies (2-OH-butyrate). Our data point out a different systolic function related to a specific metabolic activity in the two HCM phenotypic forms, with specific metabolites associated with better contractility in OHCM.

## 1. Introduction

Hypertrophic cardiomyopathy (HCM) is the most common of cardiovascular pathologies with the genetic transmission. It is caused by a variety of mutations, mainly concerning genes encoding cardiac sarcomeric proteins, and is characterized by rather heterogeneous clinical expression, with individual pathophysiology and natural history [1].

In vitro studies and animal models have shown that some sarcomeric HCM mutations are associated with subclinical impairment in contractile function and excessive sensitivity to Ca^2+^ of myofilaments. The result is an altered energy metabolism of cardiomyocytes, with an increase in ATP consumption by the cellular contractile apparatus, but with reduced efficiency of electro-mechanical coupling [2].

Metabolomics is the “omics” science that aims to analyze the profile of small molecules in a biological organism. Reflecting the exact endpoints of biological events determined by the sum of genes, RNA, proteins, and environmental factors, metabolomics provides an overall and instantaneous functional view of an organism [3]. Therefore, it is the most suitable method for studying metabolic abnormalities such as those attributed to HCM [2,4]. Accordingly, previous animal [5] and human [6] metabolomic studies showed a correlation between metabolome and both anatomical and functional echocardiographic parameters in the setting of HCM.

In this study we used gas chromatography-mass spectrometry (GC-MS)-based metabolomic analysis associated with standard and advanced echocardiography to a) highlight the metabolic specifications of the HCM, and b) distinguish and characterize the energetic substrate of both the obstructive (OHCM) and non-obstructive (NOHCM) phenotype.

## 2. Results

The anthropometric and clinical data of the study population are shown in Table 1.

We found no significant differences between the two groups regarding comorbidities or cardiovascular risk factors (Table 1).

The risk of sudden 5-year cardiac death calculated with the HCM-Sudden Cardiac Death (SCD) score [7] had a mean value of 2.76 ± 1.55%; 3 patients were carriers of implantable cardioverter-defibrillator (ICD) in primary prevention, 1 patient had been discharged with a wearable defibrillator while waiting for myectomy.

According to the literature, the HCM-SCD was higher in the OHCM group than in the NOHCM group (3.3 ± 1.8% vs. 2.1 ± 0.9%, *p* < 0.05). Patients with OHCM showed higher weight (respectively 79.44 ± 10.8 vs. 62.0 ± 10.8 kg, *p* < 0.01) and, consequently, BMI (27.9 ± 2.0 vs. 23.5 ± 3.6 kg/m^2^. *p* < 0.01) and BSA (1.9 ± 0.2 vs. 1.6 ± 0.1 m^2^, *p* < 0.01). The pharmacologic treatment did not show significant differences between the two groups.

Standard and advanced echocardiography. The whole population study showed the typical characteristics of the underlying disease, with a marked increase in the Inter-Ventricular Septum (IVS) thickness (18.7 ± 3.8 mm) and of the Left Ventricular (LV) mass measured with 3D echocardiography, both absolute (173 ± 39 g) and indexed (105.5 ± 20.2 g/m^2^), dilatation of the left atrium (Left Atrium Volume Indexed (LAVI): 52.8 ± 20.2 mL/m^2^), a normal LV Ejection Fraction (LVEF: 63.6 ± 5.2%) and reduction of the LV longitudinal function, assessed both by Speckle Tracking Echocardiography (Global Longitudinal Strain (GLS) 2D: 14.0 ± 3.6%); 3D-GLS values were comparable to those measured with two-dimensional evaluation (14 ± 3.6 vs. 14.7 ± 4.0%; *p* = 0.22).

**Figure 1:**
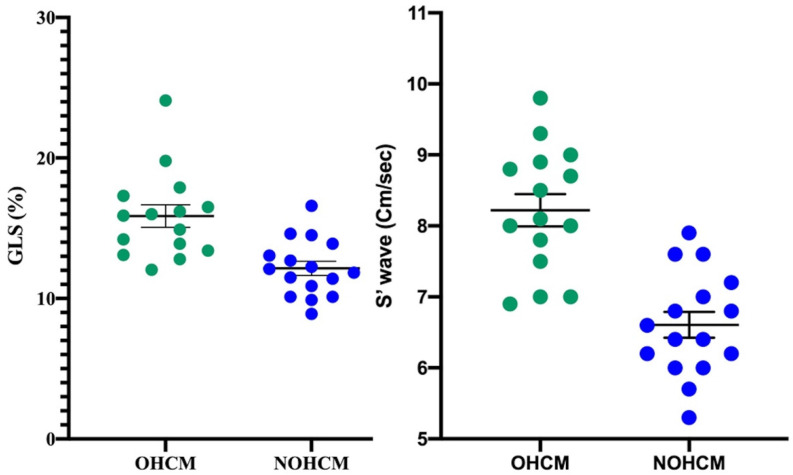
Values of TDI-derived S wave and STE-derived 3D GLS in the two Groups. OHCM (*n* = 15) vs. NOHCM (*n* = 15) Unpaired T-Test two-tailed *p*-value < 0.001 for both the comparison. GLS measurements are expressed as the absolute value.

Once divided according to the disease phenotype, our patients with OHCM presented higher values of 2D LVEF (66.0 ± 5.6% vs. 60.7 ± 2.6%; *p* < 0.01), S wave (7.6 ± 1.4 vs. 6.3 ± 0.8 cm/sec, *p* < 0.001) and 3D GLS (16.5 ± 4.3%, vs. 12.7 ± 2.7%, *p* < 0.001; Figure 1), compared to individuals with non-obstructive form; however, the first two parameters (LVEF and S wave) resulted both in the normal range.

These differences would not seem to be related to the BMI differences, as previously demonstrated in the literature [8,9]. Noteworthy, despite comparable values of diastolic function parameters, NOHCM showed higher PAPS. Standard echocardiographic parameters are reported in Table 2.

Metabolomics. To trace metabolic alterations correlated to myocardial contractility, we applied a regression analysis (Partial Least Square, PLS) between the metabolomic data (X variables) of the whole population and GLS wave (Y variable), used as a landmark continuous variable of systolic function, obtaining an excellent correlation (R^2^ = 0.82; *p* = 0.03).

**Figure 2:**
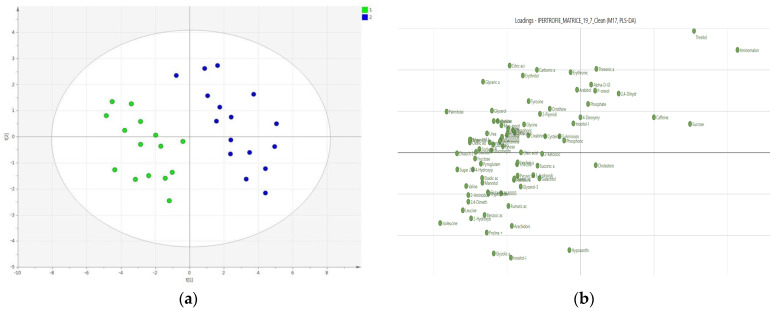
(**a**) Two-groups PLS-DA Scatter Plot of GC-MS spectra of HCM plasma samples: on the left (green dots) OHCM subjects (N = 15), on the right (blue dots) NOHCM subjects (N = 16). The figure shows the clear clustering of the two groups of subjects. ANOVA *p*-value = 0.01; (**b**) Loading scores of the PLS-DA highlighting the relative abundance of metabolites in OHCM (left inferior corner) in comparison with NOHCM (right superior corner)

The hypothesis that the 2 groups differed significantly on the basis of the phenotypic characteristics of HCM, specifically the obstructive and non-obstructive subtype, was subsequently tested; for this purpose, a 2-group PLS-Discriminant Analysis (PLS-DA) was performed, showing a clear clustering (Figure 2, Panel A) with good values of R^2^ (R^2^x = 0.889) and Q^2^ (0.622); the good quality of the model was also confirmed by the permutation test and cross-validated by ANOVA (*p* = 0.01).

The results of PLS-DA have also been reported as a loading score, which allows highlighting the weight (importance) that the variables have in separating the a priori identified classes. It is interesting to observe how it is possible to identify, even visually, a more intense metabolic activity in the OHCM group compared to that of the non-obstructive form (Figure 2, Panel B).

To rule out that the observed separation was due to differences in nutritional status, (a) the existence of a gradient along the Principal Component (PC) 1 or PC2 that followed the distribution of body weight or BMI was excluded; (b) a PLS-DA was carried out on two subgroups comparable by weight and BMI, obtaining an improvement of the model (R^2^y = 0.947; Q^2^ = 0.727), rather than a worsening, as would have been expected if the clustering was due to anthropometric differences.

**Figure 3:**
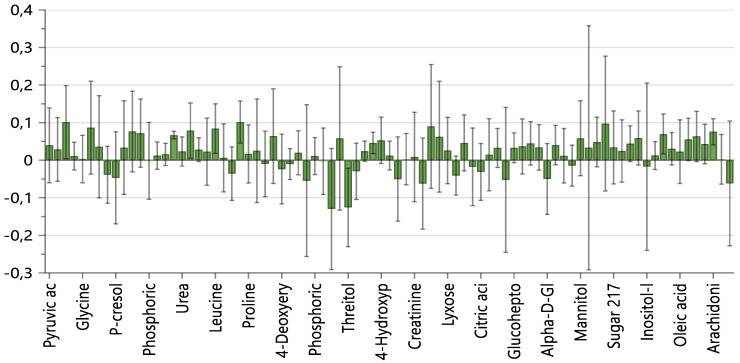
Contribution Plot of the PLS-DA showing the relative importance of metabolites in the clustering of the two Groups. Positive values: OHCM. Negative values: NOHCM.

The analysis of the loading plot and the VIPs (Variables Importance in Projection) allowed us to identify the most discriminating and expressed metabolites in the two classes (Figure 3). In the NOHCM Group, there were higher levels of threitol, aminomalonic acid, and sucrose, while the OHCM Group presented higher levels of amino acids, in particular those branched chains (BCAA), of intermediates of glycolysis (lactate) and the Krebs cycle (fumarate, succinate, citrate), of fatty acids (arachidonic acid, palmitoleic acid) and ketone bodies (2-hydroxy-butyrate).

## 3. Discussion

The present study was aimed at assessing whether metabolomics, in combination with standard and advanced echocardiographic data, was able to highlight metabolic differences underlying diverse phenotypes of HCM. The results showed: a. an excellent correlation between the contractile state, documented by S wave and GLS measurement, and the metabolomic profile in the entire population; b. a better-preserved systolic function, as demonstrated by the 2D and 3D parameters, in patients with OHCM, compared to those with NOHCM; c. a clear metabolomic clustering of OHCM and NOHCM. In this context, the most discriminating and expressed metabolites were threitol, aminomalonic acid, and sucrose in the NOHCM group, and amino acids, in particular BCAA, lactate, fumarate, succinate, citrate, fatty acids, and ketone bodies, in the OHCM group.

The alteration in energy metabolism in HCM was widely documented, by a lower value of the ratio between cardiac phosphocreatine (PCr) and adenosine triphosphate (ATP) [2], regardless of the underlying genetic mutation [10]. Moreover, the energy deficit was significantly correlated with the diastolic dysfunction but resulted independent from hypertrophy, perfusion reserve, or degree of fibrosis. Mouse models and in vitro preparations showed that the depletion of cardiac energy capacity is attributable to the inefficient use of ATP and the altered availability of intracellular calcium [4].

In recent years, metabolomics was successfully adopted to decipher the biomolecular substrate of this energy dysfunction. A mouse knock-in for the MYBPC3 gene HCM model was treated with perhexiline, a metabolic drug known to improve the myocardial production of energy. Six weeks of treatment resulted in a partial improvement in the anterior wall thickness and ventricular mass, accompanied by a substantial change of 272 metabolites, mostly involved in the cardiac energy metabolism [5].

In humans, an integrated echocardiographic and metabolomic approach was used to study 34 HCM subjects carrying the MYBPC3-Q1061X mutation, 19 mutation carriers without hypertrophy, and 20 relatives without both mutation and hypertrophy. This study found that concentrations of BCAAs, triglycerides, and ether-phospholipids were higher in HCM-bearing mutation than in controls and carriers of non-hypertrophic mutations. Furthermore, these molecules correlated with both LV hypertrophy and systo-diastolic dysfunction, suggesting their potential remodeling function in HCM [6].

The correlation between metabolome and cardiac function has been confirmed by our results. Indeed, the TDI-derived S wave and GLS, the more sensitive parameter of systolic performance, showed a strong correlation with the metabolic profile in both patient groups.

On the other hand, S wave, a well-known longitudinal dysfunction parameter [11], even though in the normal range in both the groups, was significantly lower in the NOHCM subjects compared to the OHCM group. More importantly, 3D GLS, reduced in both groups of HCM patients, was significantly more compromised in NOHCM than in OHCM subjects. As for the 3D echocardiography, the reduction of the longitudinal function in HCM patients is already well-known and has been correlated with both the degree of fibrosis and the greater susceptibility to ventricular arrhythmias [12].

The evidence of a higher LV longitudinal function in OHCM patients seems to be a particularly relevant finding of this study: despite similar LV sizes and thicknesses in both HCM groups, it could suggest a different ratio between contractile and non-contractile tissue, as previously demonstrated using cardiac Magnetic Resonance Imaging (MRI) and Positron Emission Tomography (PET) [13].

In line with this evidence, the metabolomic analysis revealed a clear difference between the two HCM subpopulations, demonstrating metabolic hyperactivity in the OHCM group, characterized by a relative hypercontractility. These findings could be explained by a relatively greater amount of metabolically active cardiomyocytes and a lesser presence of fibrotic tissue, as found by Potios et al., who identified a higher prevalence of large late gadolinium enhancement (LGE) burden on MRI and microvascular ischemia by PET in NOHCM patients [13]. This evidence, in our opinion, is relevant since it provides a molecular hypothesis for both previous and our morphological and functional echocardiographic findings.

Analyzing the VIP obtained through multivariate analysis, we found that the BCAAs in the OHCM group were the most represented molecules, in accordance with previous investigations on hypertrophied rat hearts [14] and HCM patients [6]. BCAAs, leucine, in particular, can stimulate protein synthesis, especially at the level of cardiac muscle, and demonstrate various additional effects, such as the inhibition of autophagy and the activation of mTOR [15] It is important to remember that mTOR activation is involved in the processes of hypertrophy and fibrosis of the myocardium [15]. Even the increase in lactate and intermediates of the Krebs cycle suggests a more intense energy metabolism in the OHCM group, compared to NOHCM [16,17].

Finally, in the OHCM patients the identification of increased levels of arachidonic acid and palmitoleic acid, both associated with an increased risk of ventricular arrhythmias [18,19,20], is relevant if placed in relation to the increased risk of arrhythmic death reported for these patients.

Among the molecules found to have increased in patients with NOHCM, the aminomalonic acid and threitol have recently been identified as biomarkers of pulmonary hypertension; these findings seem to be in line with the higher values of PAPS observed in our study in this group of patients. [21] For its part, aminomalonic acid has been identified in human atherosclerotic plaques [22] and found increased in patients with acute myocardial ischemia [23] and large aneurysms [24], thus suggesting a role of the substance in Cardiovascular diseases with proteolytic and/or oxidative components [24]. Moreover, aminomalonic acid has been credited with a protective effect in acute coronary syndrome [25]; nevertheless, its real biochemical significance is still not completely understood, partly because the role of the molecule in the normal metabolism is poorly recognized.

Threitol, an end-product of the xylose metabolism, is linked to the pentose-phosphate pathway through the glucuronate cycle [26]. Noteworthy, threitol was negatively correlated with LVEF in a study that evaluated metabolic fingerprints in heart failure patients before and after cardiac resynchronization therapy [27].

Sucrose, which we also found increased in patients with NOHCM, is a molecule capable of reducing the sensitivity of contractile myofilaments to Ca^2+^ and disrupting the homeostasis of this ion at the sarcoplasmic level [28], with reduced amplitude in myofilaments shortening [29].

The whole data identify a different systolic function related to a specific metabolic activity in the two HCM phenotypic forms, with better contractility in OHCM associated with a more intense metabolism, and this finding seems to confirm previous evidence of a greater amount of fibrosis in NOHCM patients (lower number of active cardiomyocytes -> lower metabolic activity -> worse systolic function), thus helping to explain the differences observed in the two forms of the same pathology.

Our study presents some limitations: the small sample size and the absence of a direct measurement of the fibrosis degree; however, 15 subjects per arm is dimed sufficient for a pilot study [30] and previous studies have already highlighted the differences between the obstructive and non-obstructive forms of HCM in terms of myocardial fibrosis and metabolic activity [13].

Moreover, our work does not pretend to be conclusive or exhaustive; rather, it would be a hypothesis-generating study and would stimulate further research on this topic in the view of a deeper knowledge of the correlation between pathophysiology and clinical phenotype of HCM.

## 4. Materials and Methods

Thirty-one patients (17 males and 14 females) affected by HCM, diagnosed according to the criteria established by the relating 2014 European guidelines [31], were enrolled in the Heart Failure Outpatients Centre of both “Azienda Ospedaliero-Universitaria” and “Azienda Ospedaliera Brotzu” of Cagliari, in the period January 2017–July 2018. Subjects with the cachectic disease, hepatic or renal dysfunction, heritable metabolic disorders, or those who had previously undergone septal myectomy were excluded from the study.

Patients were divided into two groups, based on the presence (OHCM) or absence (NOHCM) of left ventricular outflow tract (LVOT) obstruction. As indicated in the ESC HCM Guidelines [30], LVOT obstruction was defined as an instantaneous peak Doppler LV outflow tract pressure gradient ≥ 30 mmHg.

On this basis, 15 patients presented OHCM, and 16 a NOHCM phenotype (8 with a NOHCM apical form). This sample size resulted adequate to assure the minimum precision requested for a pilot study based on the work of Julious SA [31], which estimated in 12 subjects per arm the study population recommended when there is no prior information to base a sample size on.

The study was approved by the Ethics Committee of the “Azienda Ospedaliero-Universitaria di Cagliari" and was performed in accordance with the Declaration of Helsinki. Participants were informed of the purpose and methodology of the study and their written consent was obtained prior to inclusion. All patients underwent clinical evaluation, 12-lead electrocardiogram, echocardiography with the evaluation of the standard systo-diastolic parameters, and Global Longitudinal Strain (GLS) by both 2D and 3D speckle tracking echocardiography (STE). In the morning time (08–13), after a fasting night, a 4 mL heparinized venous blood sample was collected from all patients for metabolomic analysis. The risk of sudden 5-year cardiac death was calculated by the HCM-SCD score [7].

Standard echocardiography. A complete mono- and two-dimensional and Color Doppler echocardiographic evaluation was performed. The volumes and ventricular thicknesses were measured, and the left ventricle ejection fraction (LVEF) was calculated using the Simpson’s biplane method from the apical projection of four and two chambers; a value ≤ 50% has been considered abnormal. The diastolic function was evaluated by recording the velocities with the pulsed Doppler in the four apical chambers view. Using Tissue Doppler imaging (TDI), we evaluated LV longitudinal function by measuring the velocities of the mitral valve annulus. Myocardial velocity patterns were obtained from the average values measured by placing the sample volume in the basal portion of the interventricular septum (IVS) and lateral wall. Peak systolic velocity (S wave), peak velocity in proto-diastole (E’ wave), peak velocity of atrial contraction (A’ wave), and LV isovolumetric relaxation time (IVRT) were measured [32,33]. S wave was evaluated at the level of the basal segment of the IVS.

LVOT gradient was measured during a rest echocoardiography.

Raw data were also acquired using the STE technique.

3D echocardiography. The “full-volume” 3D data sets were acquired using a complete matrix array transducer (Vivid E80, GE Healthcare), positioning the probe in apical view and performing the acquisition during a brief breath interruption.

To ensure the inclusion of the entire LV within the pyramidal volume scanning and a valuable volume rate, the data sets were acquired using the "wide-angle" mode associated with a multi-beat triggered acquisition (3–4 beats) under electrocardiographic trace and during a 5–7 s apnea [34].

2D STE. The 2D STE analysis was performed using software (2D Cardiac Performance Analysis, TomTec Imaging Systems, Unterschleissheim, Germany) able to perform the analysis independently of the equipment used for the acquisition, to obtain absolute results and not vendor-specific. GLS was measured by performing a manual tracking of the endocardial border in the 3 apical views.

After STE analysis of the “frame-by-frame” LV endocardium during a cardiac cycle, the software provides the regional strain curves in each view, from which the average peak strain value is determined. The adequacy of the tracking was visually verified and, in case of tracing considered not optimal, a manual correction of the endocardial border was performed [35]. If the data obtained was not judged to be satisfactory (more than 3 segments not evaluable), the subjects were excluded from the analysis.

3D STE. 3D volumetric analysis and 3D strain measurements of the LV were performed using the 3D-STE. The “full volume” 3D data sets were analyzed using the same software as in the 2D analysis but with a specific application (4D LV analysis, Version 3.1.2, TomTec Imaging Systems, Munich, Germany).

The papillary muscles were included in the cavity of the left ventricle. Manual adjustments of the endocardial surface were performed when needed.

The software performed 3D tracking analysis throughout the cardiac cycle and LV mass was also determined.

For the 3D strain analysis, the software provided an average longitudinal strain from which the peak GLS was determined. As the software did not provide an automatic evaluation of the adequacy of the images, the accuracy of the tracking was visually assessed on the 2D images extracted from the 3D data sets. When the data was considered inadequate after manual correction the subjects were excluded from the analysis [36]; GLS was reported as an absolute value.

Metabolomic analysis. Specimens of all study participants were centrifuged at 2000 rpm for 10 min: the supernatant was transferred in Eppendorf tubes and stored at −80 °C until analysis. Plasma samples were thawed at room temperature. 100 μL of each sample were collected to form a pooled sample to use for quality control and to form an average composition sample to analyze among the others. 400 μL of plasma were treated with 1200 μL of cold methanol in 2 mL Eppendorf tubes, vortex mixed, and centrifuged 10 min at 14,000 rpm (16.9 G × 1000). 400 μL of the upper phase were transferred in glass vials (1.5 mL) and evaporated to dryness overnight in an Eppendorf vacuum centrifuge. 50 μL of a 0.24 M (20 mg/mL) solution of methoxylamine hydrochloride in pyridine was added to each vial, samples were vortex mixed and left to react for 17 h at room temperature in the dark. Then 50 μL of MSTFA (*N*-Methyl-*N*-trimethylsilyltrifluoroacetamide) were added and left to react for 1 h at room temperature. The derivatized samples were diluted with hexane (100 μL) with tetracosane (0.01 mg/mL) as internal standard, just before GC-MS analysis.

Instrumental parameters. Samples were analyzed using an Agilent 5975C interfaced to the GC 7820 (new 5977B/7890B) equipped with a DB-5ms column (J & W), injector temperature at 230 °C, detector temperature at 280 °C, helium carrier gas flow rate of 1 mL/min. The GC oven temperature program was 90 °C initial temperature with 1 min hold time and ramping at 10 °C/min to a final temperature of 270 °C with 7 min hold time. One μL of the derivatized sample was injected in split (1:4) mode. After a solvent delay of 3 min, mass spectra were acquired in full scan mode using 2.28 scans/s with a mass range of 50–700 Amu [37].

Mass Spectral deconvolution. Each acquired chromatogram was analyzed by means of the free software AMDIS (Automated Mass Spectral Deconvolution and Identification System; http://chemdata.nist.gov/mass-spc/amdis, accessed on 7 September 2021) that identified each peak by comparison of the relative mass spectra and the retention times with those stored in an in-house made library comprising 255 metabolites. Other metabolites were identified using NIST08 (National Institute of Standards and Technology’s mass spectral database) and the Golm Metabolome Database (GMD, (http://gmd.mpimp-golm.mpg.de/ accessed on 7 September 2021). Through this approach, 113 compounds were accurately identified, while 28 other metabolites were tentatively assigned relying on GMD and NIST libraries. AMDIS analysis produced an Excel datasheet that was successively subjected to chemometric analysis.

Statistical analysis. Multivariate Statistical analysis. We used the Principal Component Analysis (PCA), an unsupervised method, the Partial Least Square Analysis (PLS), a supervised regression analysis method, and the Partial Least-Square Discriminant Analysis, (PLS-DA) which uses a Y-matrix containing information about the a priori class to which the sample belongs (NOHCM or OHCM) and which is used to evaluate the statistical significance of the classification [38].

Univariate statistical analysis. Continuous variables (anthropometric, clinical-laboratory, echocardiographic data) were compared using the two-tailed T-test for non-paired samples, while the Fisher exact test was used for the categorical ones. A corrected value of two-tailed *p* < 0.05 was considered statistically significant. The analyses were performed using IBM SPSS v. 25.

## 5. Conclusions

Overall, the echocardiographic differences between NOHCM and OHCM patients seem ascribable–not only but also–to a different type of hypertrophy present in the two phenotypic forms. In turn, the metabolomic profiles, different in the 2 groups of HCM patients, a. correlate with the echocardiographic parameters of contractile function, b. could be related to a different modulation of hypertrophy/fibrosis, and c. seem to identify two different metabolic states, dependent mainly on the respective energy efficiency.

We indeed acknowledge that our results will have to be strengthened and extended with the enrollment of a larger number of patients. However, we consider the identification of specific metabolic signatures in the HCM subgroups and the related pathophysiological mechanisms as hopeful anticipation. Our data could help physicians to know better the pathophysiology and the clinical characteristics of their HCM patients. Moreover, the current results, although not conclusive, seem able to generate further research on this topic with an eminently translational approach that could lead to innovative, tailored therapies based on the identification of new molecular targets (i.e., BCAA, fatty acids), able to modify the natural history of the pathology modulating the pathways involved in its progression.

## Figures and Tables

**Table 1:** Anthropometric and clinical data of the study population.

Parameter	Whole Population	OHCM	NOHCM
Sex (M/F)	17/14	8/7	9/7
Age (years)	53.6 ± 15.3	51.6 ± 17.3	55.6 ± 17.2
Height (cm)	166 ± 9	170 ± 9	163 ± 8
Weight (kg)	71.7 ± 14.3	79.44 ± 10.8	62.0 ± 10.8 *
BMI (kg/m^2^)	25.9 ± 3.9	27.9 ± 2.0	23.5 ± 3.6 *
BSA (m^2^)	1.78 ± 0.21	1.9 ± 0.2	1.6 ± 0.1 *
Phenotype			
OHCM	15	15	0
NOHCMI	8	0	8
AHCM	8	0	8
NYHA Class			
I	19	11	8
II	9	5	4
I/II	2	1	1
II/III	1	0	1
HCM-SCD (%)	2.76 ± 1.55	3.3 ± 1.8	2.1 ± 0.9
ICD	3	3	0
CAD	0	0	0
Hypertension	4	2	2
Dyslipidemia	3	1	2
Diabetes	2	1	1
Tobacco	0	0	0
CAD Family	0	0	0
Beta-Blockers	29	15	14
ACE–I	19	10	9
ARBs	12	4	7
Ca-channel blockers	4	1	3
Statins	13	7	6
Amiodaron	3	2	1

BMI: Body Mass Index; BSA: Body Surface Area; OHCM: Obstructive Hypertrophic Cardiomyopathy; NOHCM: Not Obstructive Hypertrophic Cardiomyopathy; AHCM: Apical Hypertrophic Cardiomyopathy; ICD: Implantable Cardioverter-Defibrillator; CAD: Coronary Artery Disease; ACE-I: ACE-Inhibitors; ARBs: Angiotensin-Receptor Blockers. * *p* < 0.01 vs. OHCM.

**Table 2:** Echocardiographic data of the study population.

Title 1	Whole Population	OHCM	NOHCM	*p*(OHCM vs. NOHCM)
LV EDD (mm)	44.5 ± 5.0	44.1 ± 4.9	45.1 ± 5.1	NS
IVS. (mm)	18.7 ± 3.8	20.2 ± 3.7	17.4 ± 3.4	NS
PW (mm)	10.8 ± 2.5	11.0 ± 3.0	10.5 ± 1.9	NS
LAVI (ml/m^2^)	52.8 ± 20.2	48.9 ± 15.1	56.2 ± 24.7	NS
RV Basal EDD (mm)	35.2 ± 6.1	36.4 ± 6.7	33.5 ± 5.4	NS
RA Area (cm^2^)	19.6 ± 6.5	18.0 ± 4.7	20.0 ± 7.5	NS
LV Mass(I) (g/m^2^)	105.5 ± 20.2	112 ± 12.7	101.8 ± 23.5	NS
LV EDV(I) (mL/m^2^)	43.4 ± 10.4	46.7 ± 12.1	40.4 ± 7.3	NS
LV ESV(I) (mL/m^2^)	18.2 ± 5.4	19.5 ± 0.2	17.3 ± 4.1	NS
E/A	1.45 ± 0.43	1.24 ± 0.2	1.6 ± 0.5	NS
E/E’	13.1 ± 7.0	13.4 ± 7.0	12.8 ± 7.1	NS
PAPs (mmHg)	29.8 ± 9.3	23.5 ± 5.4	36.1 ± 13.2	<0.05
LVOT Pressure Gradient (mmHg)	28.4 ± 9.7	43.7 ± 6.1	13.2 ± 12.9	<0.01

OHCM: Obstructive Hypertrophic Cardiomyopathy; NOHCM: Not Obstructive Hypertrophic Cardiomyopathy; LV EDD: Left Ventricle End-Diastolic Diameter; IVS: Interventricular Septum; PW: posterior wall; LAVI: Left Atrium Volume Indexed; RV EDD: Right Ventricular End-Diastolic Diameter; RA: Right Atrium; LV Mass(I): Left Ventricle Mass Indexed; LV EDV(I): Left Ventricle End Diastolic Volume Indexed; LV ESV(I): Left Ventricle End Systolic Volume Indexed; PAPs: Pulmonary Artery Pressure; LVOT: Left Ventricle Outflow Tract.

## Data Availability

The datasets used and analyzed during the current study are available from the corresponding author on reasonable request because of its usage in ongoing studies.
